# Precision histology: how deep learning is poised to revitalize histomorphology for personalized cancer care

**DOI:** 10.1038/s41698-017-0022-1

**Published:** 2017-06-19

**Authors:** Ugljesa Djuric, Gelareh Zadeh, Kenneth Aldape, Phedias Diamandis

**Affiliations:** 1 Princess Margaret Cancer Centre, MacFeeters-Hamilton Centre for Neuro-Oncology Research, College Street 101, Toronto, ON M5G 1L7 Canada; 2 Laboratory Medicine Program, University Health Network, 200 Elizabeth Street, Toronto, ON M5G 2C4 Canada; 3 Department of Laboratory Medicine and Pathobiology, University of Toronto, Toronto, ON M5S 1A8 Canada

Accurate interpretation of the hematoxylin and eosin (H&E) slide has remained the foundation of pathological analysis and diagnostic medicine for over a century.^1^ For the pathologist, the H&E slide is equivalent to a high-quality patient history or physical exam. It combines art and science to help triage and guide more focused and specialized ancillary studies. Unfortunately, the perceived value of histomorphologic analysis in the era of precision medicine is diminishing in recent years due to the emergence of more contemporary and data-rich molecular studies.^2–4^ Ironically, this is no different than the scrutiny that the patient history and physical exam have faced, in light of widely available whole-body imaging technologies.^5–7^ Some have even proposed that given the exponential decrease in sequencing costs, medical assessment could effectively begin with whole-genome analysis.^8^ Here, we discuss the current state and the possible future of the H&E stain by highlighting some of its strengths and shortcomings. It may well be that the scrutiny that the H&E microscopic exam has faced in recent years^4^ is no fault of its own, but the lack of effective approaches to routinely extract more of the rich morphologic information it contains.

The H&E slide continues to be a valuable tool for pathologists and clinicians alike. For example, quite often, surgeons request urgent intra-operative pathological interpretations to help guide surgery. This clinical scenario often necessitates that an accurate diagnosis be rendered within 5–10 min. The outcome usually has huge implications for the trajectory of the remaining surgery (e.g., extent of resection, triaging additional laboratory tests). As a result, most surgeons have a strong preference for the expert opinion of highly subspecialized pathologists (e.g., from a neuropathologist for neurosurgical intra-operative consults). Until molecular or alternative analytic approaches become compatible with these acute timeframes, the H&E slide will continue to be an essential tool to help guide surgical care.

The H&E slide also has a key role in precision oncology in sub-acute settings. Technological advances now allow patients’ tumors to be globally profiled at the genomic, epigenomic, transcriptomic, proteomic, phosphoproteomic, and other -omic levels.^3, 9, 10^ This list of molecular tests, each with their own strengths and weaknesses, continues to grow. However, even with decreasing costs of sequencing, performing routine multi-platform molecular analysis on every specimen will likely not become a time-effective or cost-effective strategy in the foreseeable future. This relatively high cost of multi-omic analysis will continue to necessitate molecular triaging to help narrow testing to those most appropriate for the specific tumor type and clinical scenario. Lastly, the H&E slide still remains one of the most versatile diagnostic tools when only minute amounts of tissue, insufficient for molecular analysis, is available. Similarly, unlike bulk tissue-based molecular tests, microscopic analysis preserves important region-to-region, single-cell-level spatial information that may have significant implications for diagnostic and treatment decisions.^11, 12^ For example, even for tumors that have been analyzed at the molecular level, treatment regimens can dramatically change when specific microscopic features are noted (e.g., lymphovascular invasion, metastatic foci, elevated mitotic activity,^13^ tumor morphology).^14^ Therefore, there are many compelling reasons to retain the H&E exam as a non-overlapping and essential tool in our growing precision oncology toolbox.

**Fig. 1:**
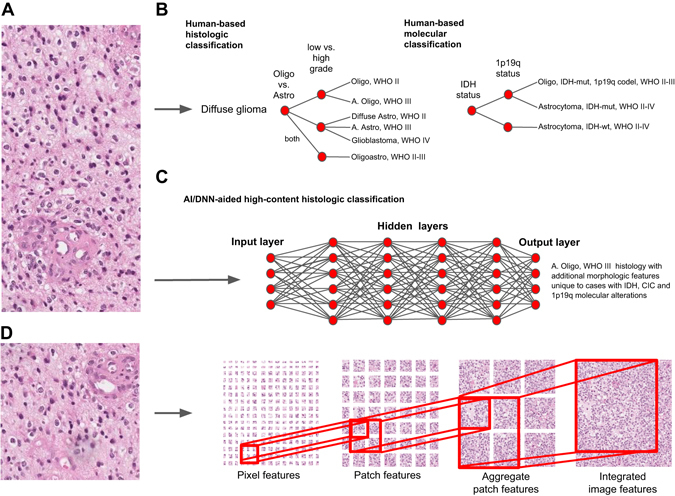
Diagnostic decision-making algorithms in pathology. **a** Digital image of a H&E stained slide showing classic histologic features of an anaplastic oligodendroglioma, WHO grade III. **b** Unaided human-based classification algorithms utilize simple and highly reproducible decision tree-based approaches. Even with new more objective molecular tools, the need for highly uniform and reproducible diagnostic reporting limits the number of alterations that can be extracted from data-rich molecular data sets. **c**, **d** Machine learning-based approaches may allow for multi-parametric feature extraction and aggregation that permit even subtle but reproducible groups of features to be integrated into classification schemes. DNNs may thus tolerate reliable extraction of a larger number of unappreciated features that can be correlated with specific entities, outcomes, and molecular changes. *IDH* isocitrate dehydrogenase, *IDH-mut* IDH-mutated, *IDH-wt* IDH-wildtype, *CIC* capicua, *Oligo* oligodendroglioma, *Astro* astrocytoma, *Oligoastro* oligoastrocytoma, *WHO* World Health Organization malignancy grading scheme

Perhaps a major limitation of the H&E slide in the era of “big-data” is the unassisted human interpretation currently used for analysis. To promote consistency and objective inter-observer agreement, most pathologists are trained to follow simple algorithmic decision trees that sufficiently stratify patients into reproducible groups based on tumor type and aggressiveness (Fig. 1a–b). For example, in the most common group of brain tumors known as diffuse gliomas, the pathologist first begins by examining nuclear morphology to decipher a cell of origin (e.g., astrocytoma vs. oligodendroglioma). Once this first decision is established, the pathologist next assigns a degree of malignancy based on the presence of mitotic activity, tumor necrosis, and vascular proliferation (WHO grade II–IV). Even with these simplified algorithms that focus on binary and sufficiently different features, inter-observer discordance still persist, even among sub-specialists.^3, 15, 16^ This diagnostic uncertainty has promoted liberal and widespread use of costly molecular testing to differentiate between seemingly histologically indistinguishable lesions.^2, 3, 17, 18^ Similarly, in efforts to maintain diagnostic objectivity, other potential prognostic and therapeutic morphologic biomarkers, such as foci of tumor-infiltrating lymphocytes and fibrotic tumor reaction, are often omitted. Indeed, even in the molecular era, the unassisted physician still largely relies on simple decision tree approaches that utilize only a small fraction of available -omics knowledge (Fig. 1b). This simplified, technology-free and unaided approach to histopathology is thus not maximizing the complex morphological information present for optimal patient management.

In an increasing number of social and clinical scenarios, a form of artificial intelligence (AI) known as deep neural networks (DNNs) is proving to be a valuable tool for the generation and implementation of complex multi-parametric decision algorithms.^19–25^ With appropriate training, DNNs can match and even outperform expert-level decision-making in strategic board games such as chess and Go.^19, 20^ More recently, DNN-based image analysis of skin lesions has proven capable of yielding diagnostic interpretations with accuracy similar to board-certified dermatologists!^21^ With the recent improvement in high-throughput whole-slide scanning technologies and image computing, DNNs are poised to make H&E slide analysis and classification a new source of large biological data sets for precision oncology.^24, 26–29^

Unlike simplified algorithms pathologists are trained to use, DNNs deconstruct images into pixels and sequentially aggregate them to form shapes (e.g., lines) and reproducible features that represent distinct diagnostic patterns (Fig. 1c–d). When given enough annotated training images, even subtle feature differences between clinically significant groups can be theoretically extracted and used to classify future cases.^23, 26^ With robust clinically and genomically well-annotated training sets, minute differences not apparent to the human observer may even be used to aid classification and predict likelihood of specific molecular alterations and prognosis^26^ (Fig. 1c). Such image-based exercises, as we have seen, are ideally suited for deep learning^21^ and could provide significant time and cost-saving measures when prioritizing molecular testing in precision oncology. They could also significantly improve pathologists’ productivity by highlighting actionable morphologic features (e.g., mitotic figures, lymphovascular invasion) that are time-consuming to identify by the unaided human eye.^24, 28, 29^

Digital revitalization of histomorphology is already showing promise. For example, Dong et al. recently showed that upwards of 22 different morphologic nuclear features (e.g., size, shape, texture) could be extracted and used to train classification models to reliably differentiate between neoplastic (ductal carcinoma in situ) and non-neoplastic (ductal hyperplasia) breast lesions.^23^ Such studies highlight how machine learning can extract novel and multi-parametric morphologic features not readily accessible by the human eye.^23, 30^ In addition to refining current diagnostic criteria, a recent study by Yu et al. showed that automated computer-based image analysis of the non-small cell lung cancers found in The Cancer Genome Atlas (TCGA) could identify novel morphologic features that predict survival.^26^ Integration of DNN-based analyses will likely continue to push the boundaries of clinically relevant morphologic information not found in our current human-derived pathologic criteria.^28, 31^ More recently, the use of DNNs to scan for metastatic tumor foci in lymph nodes achieved substantially lower false-negative rates when compared to pathologists (from 26.8 to 7.6%).^24, 29^

Automation and routine AI-based analysis of the H&E slide to include these milestones would provide immediate benefits to patient care. For example, robustly trained DNNs may assist general pathologists in providing subspecialist-level expertise for intra-operative consultations. Integration of AI/DNNs into laboratory medicine could eventually shift training and the role of future pathologists toward “big-data” information specialists.^22^ Such an evolution would help address the shortage of pathologists and improve consistency in reporting.

Similarly, automating AI-assisted histologic analysis in non-emergent settings would likely also improve diagnostic workflow. Currently, there are numerous “practical” delays in rendering diagnoses. There is usually a day lost between generation of an H&E slide and delivery to a pathologist or clinical fellow for review. Once analyzed, the pathologist generates a differential diagnosis and triages appropriate immunohistochemical tests to narrow diagnostic considerations. This usually takes an additional 2–3 days. Only then are appropriate diagnostic and confirmatory molecular tests considered. This linear series of steps, and the need to revisit cases multiple times, creates significant delays in rendering diagnoses and valuable molecular information to clinicians for personalized patient care. Often, a multitude of unnecessary molecular tests are liberally and prematurely ordered in attempts to avoid such delays. Implementation of DNN-based image analysis could allow a preliminary digital diagnosis to be provided and initiation of appropriate immunohistochemical stains and molecular tests before the slides even reach the pathologists’ microscope. Automated selection of lesional regions of interest and initiation of relevant multi-omics testing could even be initiated minutes after the initial slide is generated!

Recent large-scale immunohistochemical efforts are also generating massive amounts of protein-based histologic information across different tissue and cancer types.^11, 12^ These valuable and publicly available resources are ideally suited to train DNNs to incorporate follow-up studies into their diagnostic algorithms. Machine-driven decisions could therefore provide improved and optimized immunohistochemical approaches that lead to rapid and cost-effective diagnostic convergence among various other morphologic considerations.

This revised workflow would significantly expedite pathology sign-out by having pathologists function as analytic experts that unify clinical, morphologic, and molecular information into an integrated diagnosis.^22^ Robust training of DNNs with a large number of molecularly confirmed cases, such as those found in TCGA, may also identify subtle morphologic features that better predict presence/absence of molecular alterations.^32^ This could provide significant cost savings for pathology departments without affecting patient care. Similarly, training DNNs with images from known responders and non-responders to specific treatments (e.g., immunotherapy) may help to better stratify patients for appropriate future personalized and precision-based clinical trials. For example, the presence of tumor-infiltrating myeloid (TIM) cells are thought to promote invasion and immunosuppression in glioblastomas.^33^ Their presence in the tumor microenvironment may be an important cause of treatment failure to both conventional and immune-based therapies.^34, 35^ Prompt and reliable quantification of TIM in gliomas may thus provide vital information to stratify a patient to combined tripartite immune therapies that include colony stimulating factor 1 inhibitors.^34^ Alternatively, H&E-based deep learning may uncover specific TIM density thresholds that, when exceeded, suggest that immune-based therapies will likely be ineffective and signal prioritization of patients to other therapeutic strategies. Similarly, neutrophilia in glioblastomas have been associated with release of elastase that promotes brain invasion and malignancy.^36, 37^ High-content image analysis that quantifies these additional immune cell parameters may help predict patients with well-circumscribed tumor borders. Such patients could be good candidates for de-escalation therapy^38^ following gross total resection. AI-based discovery of robust morphologic surrogates of genetic alterations (i.e., BRAF V600E^39–42^ and IDH^43, 44^ mutations) may even become objective stand-alone inclusion criteria that could grant patients early enrollment in precision-based clinical trials as they await confirmatory molecular testing (Fig. 1c).

Lastly, quality assurance is becoming an increasingly relevant component in precision oncology. Most cancer centers encourage, or even require, regular peer-review and consensus on all oncologic diagnoses. An online cloud-based DNN-based image analysis tool may thus provide a fast and reliable way for pathologists at small centers to receive timely and cost-effective second opinions and consensus.

Thankfully, unlike many other emerging molecular technologies, AI-based diagnostics presents a relatively cost-effective addition to the current workflow with a low financial barrier of entry for most institutions. Recent digital pathology educational initiatives have helped equip most pathology departments with whole-slide scanning technologies that can digitize slides in a standardized high-resolution format.^45, 46^ In fact, improvements in throughput (1 slide/minute) and quality, coupled with the decreasing cost of data storage, are now at a point where some pathology departments are considering archiving their complete slide libraries digitally.^46–48^

Similarly, many technical concerns of computer-assisted diagnostics have now also been adequately addressed. For example, there have been concerns that variations in imaging quality resulting from difference in staining protocols, technical handling, and scanner setting could introduce “non-biological” batch effects that can significantly compromise the performance of computer-driven morphologic analysis and classification accuracy. Fortunately, these concerns have now also been largely addressed with quality control algorithms that can carry out automated color normalization to reduce batch-related variance to insignificant levels.^49–52^ Introduction of random perturbations to the brightness and color saturation levels of training images allows AI-based algorithms to effectively learn color-invariant features for classification.^29^ Such simple image-augmentation procedures allow for cross-institutional sharing and pooling of digital resources that could allow DNNs to handle even rare and highly variable tumor types. Similarly, they allow access to freely available and multi-omic data sets (e.g., TCGA) to carry out large-scale morpho-genomic corrrelations.^50^

As scanners can now routinely handle batches of 400 slides, it is conceivable that the current throughput of scanning could allow for routine digitization and analysis of every slide produced in a large proportion of pathology departments. Even if additional capital and personnel investment is necessary, image-based analysis is already making a compelling case as a cost-effective tool to reduce pathology workload and excessive confirmatory tests to a more sustainable level. In one study, for example, DNNs could effectively highlight slides with cancer for pathologists and safely exclude 30–40% of slides containing only normal tissue.^25^

Despite technological advances and criticisms, the physical exam has endured the test of time to remain a powerful, cost-effective, and adaptable diagnostic tool that contributes to strengthen the patient–physician bond, trust, and ultimately patient care.^53^ The H&E slide provides many of the same positive sentiments to pathologists and represents the origins of precision-based diagnostics and medicine.^1^ DNN-based technologies are now poised to revolutionize how pathologists use the H&E slide by helping physicians extract unprecedented and colossal amounts of objective and multiparametric morphologic information. Although recent studies highlighted give reasons for optimism, this field is still in its infancy. Integration of this new technology into diagnostic workflows will require pathologists to continue to practice their mastery of the microscopic exam to oversee and approve machine-based interpretations. Such an evolution will allow the H&E slide to remain a pivotal component to the multi-omics approach to personalized and precision oncology.
